# Real-time estimation and forecasting of COVID-19 cases and hospitalizations in Wisconsin HERC regions for public health decision making processes

**DOI:** 10.1186/s12889-023-15160-6

**Published:** 2023-02-17

**Authors:** Srikanth Aravamuthan, Juan Francisco Mandujano Reyes, Brian S. Yandell, Dörte Döpfer

**Affiliations:** 1 Department of Medical Sciences, University of Wisconsin, Madison, WI USA; 2 Department of Statistics, University of Wisconsin, Madison, WI USA

**Keywords:** COVID-19, Forecasting, Statistical modeling, Prediction uncertainty, Bayesian inference

## Abstract

**Background:**

The spread of the COVID-19 (SARS-CoV-2) and the surging number of cases across the United States have resulted in full hospitals and exhausted health care workers. Limited availability and questionable reliability of the data make outbreak prediction and resource planning difficult. Any estimates or forecasts are subject to high uncertainty and low accuracy to measure such components. The aim of this study is to apply, automate, and assess a Bayesian time series model for the real-time estimation and forecasting of COVID-19 cases and number of hospitalizations in Wisconsin healthcare emergency readiness coalition (HERC) regions.

**Methods:**

This study makes use of the publicly available Wisconsin COVID-19 historical data by county. Cases and effective time-varying reproduction number $R_t$ by the HERC region over time are estimated using Bayesian latent variable models. Hospitalizations are estimated by the HERC region over time using a Bayesian regression model. Cases, effective Rt, and hospitalizations are forecasted over a 1-day, 3-day, and 7-day time horizon using the last 28 days of data, and the 20%, 50%, and 90% Bayesian credible intervals of the forecasts are calculated. The frequentist coverage probability is compared to the Bayesian credible level to evaluate performance.

**Results:**

For cases and effective $R_t$, all three time horizons outperform the three credible levels of the forecast. For hospitalizations, all three time horizons outperform the 20% and 50% credible intervals of the forecast. On the contrary, the 1-day and 3-day periods underperform the 90% credible intervals. Questions about uncertainty quantification should be re-calculated using the frequentist coverage probability of the Bayesian credible interval based on observed data for all three metrics.

**Conclusions:**

We present an approach to automate the real-time estimation and forecasting of cases and hospitalizations and corresponding uncertainty using publicly available data. The models were able to infer short-term trends consistent with reported values at the HERC region level. Additionally, the models were able to accurately forecast and estimate the uncertainty of the measurements. This study can help identify the most affected regions and major outbreaks in the near future. The workflow can be adapted to other geographic regions, states, and even countries where decision-making processes are supported in real-time by the proposed modeling system.

## Background

The COVID-19 (SARS-CoV-2) global pandemic was first reported in Wuhan, China in December 2019, and reached the United States in mid-January 2020 [1]. By February of 2020 community level transmission was already a concern in the U.S [1]. The spread of the COVID-19 and surging number of cases across the US have resulted in overtaxed healthcare systems. As of November 1, 2021, more than 46 million people in the United States have been infected with SARS-CoV-2, almost 750,000 have died, and approximately 50,000 are hospitalized daily [1, 2].

Different efforts to mitigate the negative impacts of the pandemic using public data have been done. Many of them have been reported in a dashboard format with the objective of display real-time updates [3–7]. However, limited availability and questionable reliability of data make this reporting difficult [8]. Therefore, outbreak prediction and resource planning are in need of accurate modelling approaches. Such prediction models provide tracking of the pandemic stages with the aim to improve decision-making process by the public health authorities.

Autoregressive and time series approaches are a common feature for many prediction and forecasting strategies [9, 10]. During the early stages of the pandemic, simpler models used deterministic parameters for fitting a curve to the raw data [11, 12]. Other approaches used compartmental models assuming homogeneous mixing of the population or closed population while ignoring covariates affecting the infections dynamics [13–19]. Specifically, Susceptible-Exposed-Infectious-Removed (SEIR) model have been used to describe the spread of the virus and compute the number of infected, recovered, and dead individuals based on the number of contacts, probability of disease transmission, incubation period, recovery rate, and fatality rate [20]. SEIR models and its variants have been applied to different scenarios including measles in Niger, pertussis in the United States, and syphilis in China as well as estimating current COVID transmission and forecasting case counts [20–26].

Nevertheless, the SEIR modeling framework are not consistent with all COVID-19 pandemic features and the outbreak dynamics are subject to various parameters where information is not available or incomplete [27]. Moreover, movement between locations, mixing within location, age structure, and super-spreader events must be explicitly included into the model [28]. Model specification can be challenging since transmission rates are generally defined to be constant and does not account for all possible transmission pathways. Poor data input, wrong modeling assumptions, high sensitivity of estimates, and lack of incorporation of epidemiological features are a few of the issues regarding epidemic forecasting [29]. SEIR models do not consider the reporting delay of cases and hospitalizations in the predictions. Therefore, estimates or forecasts are subject to uncertainty and imperfect accuracy when projecting such data.

An empirical Bayes implementation has been shown to produce accurate forecasts [30, 31]. Furthermore, Bayesian approaches has been shown to be flexible where a model can estimate the cross-sectional distribution for covariates and then forecast metrics for time series data [32]. However, more sophisticated modeling approaches are required to make the pandemic prediction and forecasting feasible [27].

For all modelling approaches aimed at forecasting SARS-CoV-2 cases and hospitalizations, uncertainty is propagated from all inputs [33]. These sources of variation include delay between positive test and hospitalization, reporting delay between symptomatic and positive test, as well as incubation period and generation time [34, 35]. Predictions accounting for the uncertainty in the final parameter estimates are needed.

The study aims to apply, assess, and automate a workflow for the real-time estimation and forecasting of COVID-19 cases and hospitalizations using Wisconsin HERC regions data. Positive cases are corrected by data cleaning and time series smoothing using generalized additive models. The time-varying effective reproduction number is estimated by using a Bayesian latent variable model [33, 36]. Finally, hospitalization admissions are estimated using a Bayesian generalized non-linear multivariate multilevel model. The study is designed, developed, and implemented to anticipate future surges and prevent COVID-19 outbreaks in Wisconsin using early prediction and accurate forecasting. Such tools help public health officials and other decision-makers to implement preventive strategies and to improve response measures regarding the growth and spread of COVID-19 without overwhelming healthcare systems.

## Methods

### Data

**Fig. 1:**
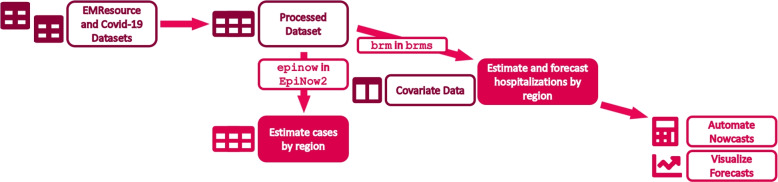
Workflow for estimating and forecasting cases and hospitalizations by Wisconsin HERC region

The Emergency Medicine (EM) Resources and COVID-19 Historical Data by County for Wisconsin datasets are used to estimate the cases and hospitalizations [37]. The datasets include information including name of the geographic boundary, date and time when data have been last updated and published, number of people who have tested negative for SARS-CoV-2 RNA, the number of confirmed cases of COVID-19, the number of hospital admissions with confirmed COVID-19. All results from diagnostic and confirmatory tests to detect the RNA of SARS-CoV-2 virus causing COVID-19 for Wisconsin residents are reported to the Wisconsin Department of Health Services (DHS), then entered and aggregated by the Wisconsin Electronic Disease Surveillance System (WEDSS). COVID-19 cases are defined by the Centers for Disease Control and Prevention (CDC) [38]. If a person had more than one positive result based on the standard diagnostic test, such cases are counted as a confirmed case only once. The data are preprocessed to clean and validate the dataset before estimating the effective $R_t$ and cases. The missing values are imputed using linear interpolation by fitting a line between last and next observed values. The monotonicity issue of ascending cumulative cases because of removing or reassigning cases after receiving new information is remedied by retroactively correcting for decrease in cumulative cases. The number of new positive cases is smoothed using generalized additive models for test positivity by date based on a quasibinomial family [39, 40]. The corrected positive cases are calculated by taking the product of the new positive cases and a correction factor described below adjusting for underreporting [41–43]. The correction factor is specific to the Wisconsin dataset and defined as the ratio of the smoothed test positivity for a given county on a given day and the 2.5 percentile of smoothed test positivities for all counties on the same date. The data are filtered to include the time period from September 20, 2020 to December 6, 2020 corresponding to a COVID-19 surge in Wisconsin. The data are aggregated by healthcare emergency readiness coalition (HERC) regions of a core group of hospitals and healthcare organizations, local and tribal public health agencies, state, regional, and local and tribal emergency management, and emergency medical services, as well as additional members. Wisconsin has seven HERC regions that support communities during disasters and other health-related crises by allocating resources [44]. The positive cases corrected for missing values, monotonicity, and test positivity are directly used to estimate the cases, effective reproduction number, and hospitalizations as illustrated in Fig. 1.

### Estimated cases by date of infection and time-varying effective reproduction number $R_{t}$

The time-varying effective reproduction number $R_t$ is estimated using a Bayesian latent variable model from a range of open-source tools and current best practices [33, 36]. The model assumes that limited time series data for case counts are available by date of symptom onset and instead uses date of report. The data are then imputed to case counts by date of infection using a frequency distribution for the reporting delay and incubation period as described below. Binomial upscaling was used to increase the estimated numbers of case onsets close to the present to account for right truncation. Time-varying estimates of the reproduction number are estimated by date of infection including uncertainty from a frequency distribution for the generation time as described below. The time-varying reproduction number is forecasted 7-days forward in time to remain constant. The $R_t$ and the corresponding case forecasts are implemented in the R package *EpiNow2* [45].

The initial number of infections is estimated using a prior distribution based on the initial number of cases. Previous infections are weighted by the generation time probability mass function (*w*), summed, and multiplied by the $R_t$ for the infections at time *t* ($I_t$). A log normal prior distribution with mean 1 and standard deviation 1 is used for the reproduction number ($R_0$). The infection trajectories are used to calculate mean reported cases ($D_t$) for each incubation period and report delay distribution (convolved into $\xi$). Observed reported cases ($C_t$) are generated from a negative binomial distribution with a mean $D_t$ multiplied by a day of the week effect ($\omega _{(t \, mod \, 7)}$) and an overdispersion parameter $\phi$. An exponential prior distribution with mean 1 is used to generate the overdispersion parameter $\phi$. Lastly, randomness is introduced using an approximate Gaussian process with a squared exponential kernel (*GP*) [33, 36]. The equations for estimating the time-varying reproduction number $R_t$ and nowcasting reported infections are defined below.

$$\begin{aligned} R_{t}\sim & {} R_{t-1} \times \mathrm {GP} \\ I_{t}= & {} R_{t} \sum _{\tau } w_{\tau } I_{t-\tau } \\ D_{t}= & {} \sum _{\tau } \xi _{\tau } I_{t-\tau } \\ C_{t}\sim & {} \mathrm {NB}\left( D_{t} \omega _{(t \, mod \, 7)}, \phi \right) \end{aligned}$$

A log-normal distribution is fit to estimate the reporting delay with appropriate uncertainty using 100 subsampled bootstraps of a log-normal distribution of $\mu =6$ and $\sigma =1$ [41–43]. For computational purposes, the maximum allowed delay is set to be 30 days. Each date is rounded to the nearest day and truncated to the maximum observed delay accounting for left and right censoring. The generation time follows a gamma distribution with a mean of 3.6 days and a standard deviation of 3.1 days and the incubation period follows a log-normal distribution with a mean of 1.6 days and a standard deviation of 0.418 days [34, 35]. 

The time-series is truncated to include the last 28 days of data and a rolling average for the prior of 7 days is taken based on reported cases. The effective $R_t$ is fitted every week and forecasted over a 1-day, 3-day, and 7-day time horizon, then transformed into a case forecast. The parameters of the Gaussian process kernel are estimated during model fitting. The prior on the magnitude is standard normal. Each timeseries is fit independently using Markov-chain Monte Carlo (MCMC). Two chains are used with a warmup of 200 each and 1000 samples post warmup. Convergence is assessed using the R hat diagnostic.

### Estimated hospitalization by date of admission

The number of hospitalization admissions is estimated using a Bayesian generalized non-linear multivariate multilevel model implemented in the R package *brms* [46]. A log-normal distribution is fit to estimate the hospitalization ($H_t$). A flat prior for each region $\mu _t$ is used. The model is fit with a smoothing term for the date $\psi _t$ to estimate the local linear trend and a random effect term for day of the week $\gamma _t$ to estimate the seasonal pattern. The prior on the random effect term is defaulted to student-t with a mean of 0, a standard deviation of 2.5, and 3 degrees of freedom. For computational purposes, the time-series is truncated to include the last 28 days of data by date of admission and the hospitalization is fitted every week and forecasted over a 1-day, 3-day, and 7-day time horizon. Each timeseries is fit independently using Markov-chain Monte Carlo (MCMC). Two chains are used with a warmup of 1000 each and 1000 samples post warmup. Convergence is assessed using the R hat diagnostic. The equations for nowcasting hospitalization admissions are defined below.

$$\begin{aligned} H_{t}= & {} \mu _t + \gamma _t + \varepsilon _t \\ \mu _t= & {} \mu + \psi _t + \eta _{\psi ,t} \end{aligned}$$

### Computed coverage probability of the Bayesian credible interval

The Bayesian credible interval is the range of values from the posterior distribution containing the credible probability, given the observed data at a specific level. The coverage probability is the proportion of values for $R_t$, cases, and hospitalizations that are contained in the forecasted credible interval [47]. The $R_t$, cases, and hospitalizations are forecasted over a 1-day, 3-day, and 7-day time horizon using the last 28 days of data, and the 20%, 50%, and 90% Bayesian credible intervals of the forecasts are calculated. The credible probability of the $R_t$ and cases is the proportion of the Bayesian credible interval that contains their respective estimated median values. The estimated median values for $R_t$ and cases are computed for the dates of interest using the model fitted the data 14 days later. In contrast, the credible probability of the hospitalizations uses their observed values instead of the estimated median values.

### Software requirements

All analyses are performed using the statistical programming language *R* version *4.0.3* [48]. The analyses include estimating the effective $R_t$, cases, and hospitalizations by date of infection as well as automating the workflow and building an interactive for the effective $R_t$ time series data by Wisconsin HERC regions. The EM Resources and COVID-19 Historical Data by County datasets are retrieved from the Wisconsin Department of Health Services and cleaned prior to calculating the adjusted case counts and plotting the effective $R_t$, cases, and hospitalizations using the R package *tidyverse* version *1.3.0* [49]. All real-time case counts and time-varying epidemiological parameters are computed by means of the R package *EpiNow2* version *1.1.0* [45] implementing a Bayesian latent variable approach using Stan. Correcting reported cases for under-reporting is performed using the R package *mgcv* version *1.8-33* [50]. All real-time hospitalization counts are computed by means of the R package *brms* version *2.15.0* [46, 51] fitting a Bayesian regression model using Stan. All visualizations are created using the R package *ggplot2* version *3.3.2* [52] and translated to add interactivity by means of the R package *plotly* version *4.9.2.2* [53] powered by the JavaScript library *plotly.js*. The two R packages are used to apply the consistent and expressive interface for exploring statistical summaries across Wisconsin counties. Geofaceting of the HERC regions map for the state of Wisconsin is performed using the R package *geofacet* version *0.2.0* [54]. All analyses described above are updated daily for each region and the visualization is implemented as a webpage: https://data-viz.it.wisc.edu/cases-r-hosp-geofacet-wi-region/ [55].

## Results

**Fig. 2:**
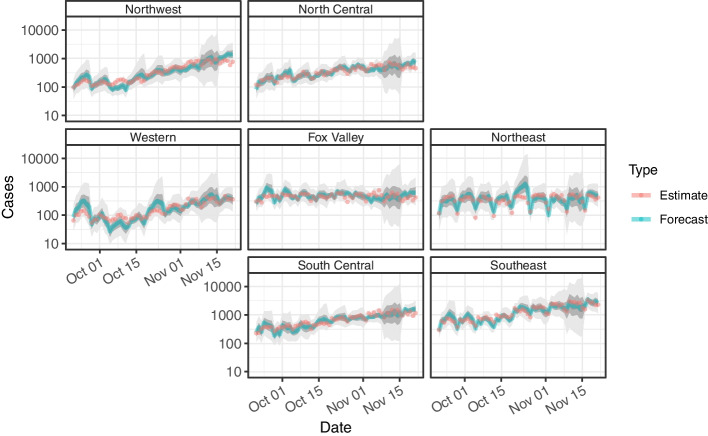
Geofacet of time series data for estimated and forecasted cases. Geofacet of time series data for estimated and forecasted cases by Wisconsin HERC region generated from September 20, 2020 to December 6, 2020

**Fig. 3:**
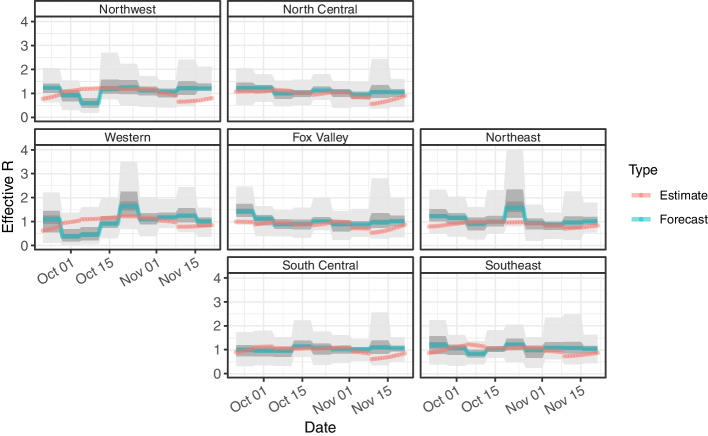
Geofacet of time series data for estimated and forecasted effective $R_t$. Geofacet of time series data for estimated and forecasted effective $R_t$ by Wisconsin HERC region generated from September 20, 2020 to December 6, 2020

**Fig. 4:**
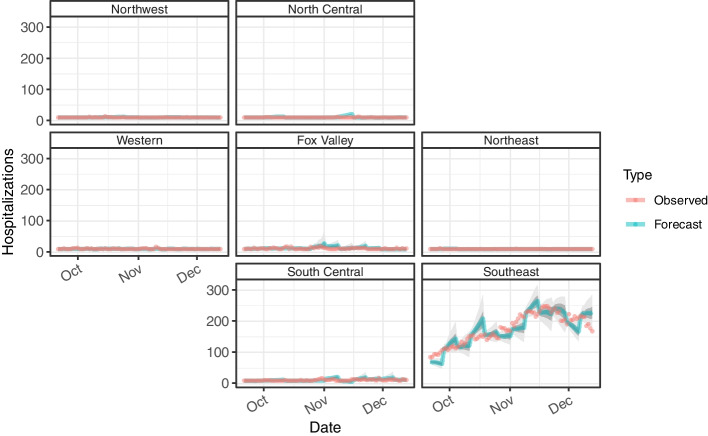
Geofacet of time series data for estimated and forecasted hopitalizations. Geofacet of time series data for estimated and forecasted hopitalizations by Wisconsin HERC region generated from September 20, 2020 to December 6, 2020

All models are fitted every week and forecasted over a 1-day, 3-day, and 7-day period during the peak of the epidemic from September 20, 2020 to December 6, 2020 (Figs. 2, 3 and 4). The plots are geofaceted such that the panels in the grid are arranged by the geographic orientation of the regions. The forecasted median values of the measures are encoded by the blue lines and the estimated median value of the measures for the respective dates are encoded by the red points. The 20%, 50%, and 90% Bayesian credible intervals of the forecasts are depicted by the dark gray, medium gray, and light gray respectively. For cases, the respective coverage probabilities is greater than all three credible levels for all three forecasts (Fig. 2). The 7-day period (20% CrI: 0.324, 50% CrI: 0.707, 90% CrI: 0.986) performs slightly better than both the 1-day period (20% CrI: 0.302, 50% CrI: 0.683, 90% CrI: 0.968) and the 3-day period (20% CrI: 0.302, 50% CrI: 0.677, 90% CrI: 0.979). Overall, the credible intervals underestimates the coverage where the forecasts are more accurate than expected such that reported intervals are much wider than necessary.

Similarly, for effective $R_t$, the respective coverage probabilities is greater than all three credible levels of the forecast for all three forecasts (Fig. 3). The 7-day period (20% CrI: 0.263, 50% CrI: 0.705, 90% CrI: 0.970) overall performs slightly better than the 1-day period (20% CrI: 0.254, 50% CrI: 0.698, 90% CrI: 0.968) and 7-day period (20% CrI: 0.270, 50% CrI: 0.698, 90% CrI: 0.968). Likewise, the credible intervals underestimate the coverage for effective $R_t$ similar to cases.

For hospitalizations, a Bayesian generalized non-linear multivariate multilevel model is fitted using region and day of the week and smoothing over date. The best model is determined by coverage probability of the 90% credible interval of the forecast. The respective coverage probabilities is greater than the 20% and 50% credible interval of the forecast for all three forecasts (Fig. 4). However, the 1-day and 3-day period underperforms the 90% credible interval. Only the 1-day period (20% CrI: 0.631, 50% CrI: 0.762, 90% CrI: 0.905) outperforms all three credible level of the forecast and performs considerably better than the 3-day period (20% CrI: 0.575, 50% CrI: 0.702, 90% CrI: 0.845) and 7-day period (20% CrI: 0.563, 50% CrI: 0.689, 90% CrI: 0.847). Note, the vast majority of hospitalizations are admitted in the Southeast HERC region during the time period of interest. The addition of other population-associated measures including cases by age groups generated slight worse results based on coverage probability and omitted from further analysis.

**Fig. 5:**
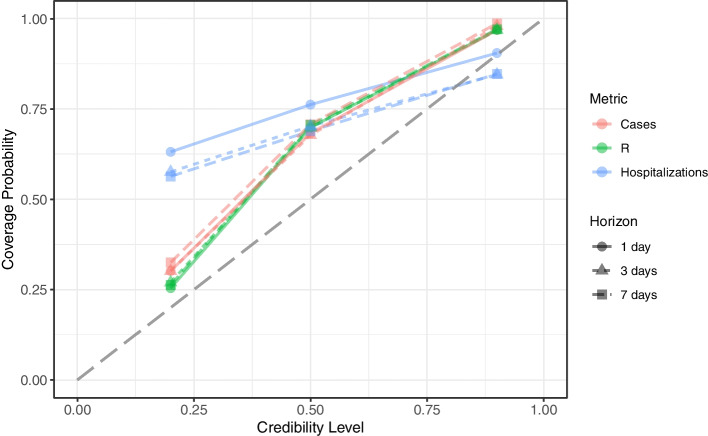
Coverage probability of time series data for estimated and forecasted cases, effective $R_t$, and hopitalizations. Coverage probability of time series data for estimated and forecasted cases, effective $R_t$, and hopitalizations by Wisconsin HERC region generated from September 20, 2020 to December 6, 2020

However, the 20%, 50%, and 90% credible intervals underestimate the true coverage probabilities for cases, $R_t$, and hospitalizations and 1-day, 3-day, and 7-day horizons except for two of the forecasts. The underestimation of the coverage probabilities by the credible intervals correspond to the data points above the dashed gray line in Fig. 5. Only the 90% credible interval overestimate the true coverage probabilities for only hospitalizations at 3-day and 7-day horizons and corresponds to the two data points below the dashed gray line in Fig. 5. Therefore, the question of uncertainty quantification should be restated as the frequentist coverage probability of the Bayesian credible interval based on the observed data. Consequently, a coverage probability of 0.90 on the y-axis corresponds to a credible level of 0.80 for cases on the x-axis interpolated between the credible levels of 0.50 and 0.90.

## Discussion

Publicly available data are used for real-time estimation of key metrics and visualizations to support decision making processes in public health [3–7, 56]. Traditionally, compartment models are used for modeling infectious diseases and many public health decision making tools do not use real-time data or require user-defined inputs of key model parameters [13–19, 57–60]. Other decision tools provide real-time estimates of epidemic parameters such as the cumulative number of infections, but no uncertainty measure [13, 14, 59].

Reliable forecasts are necessary to support health care organizations and public health agencies before health-related crises by allocating essential resources [61–64]. We used a Bayesian framework to generate robust estimates for small, real-time data without having any extra prior knowledge [63, 65]. Given a Bayesian framework, we do not make assumptions about the population under study. On the contrary, deterministic models assume homogeneous mixing of the population or closed population with no migration, births, or deaths from causes other than the epidemic [66]. The parameters in traditional compartmental models do not readily quantify the uncertainty of the model parameters. Although no model can perfectly forecast the future, the workflow provides accurate estimates for use in forming public policy [66].

The Bayesian latent variable model is based on the best practices to measure the effective reproductive number $R_t$ using a range of open-source tools implemented in *EpiNow2* [33, 36, 45]. The tool is used to explore the sensitivity of $R_t$ estimates to different data sources and the transmission of COVID-19 for different population sub-groups in England [67]. Additionally, the implementation is used to provided probabilistic real-time forecasts of COVID-19 cases and deaths in Germany and Poland during the second wave [68].

The Bayesian latent variable model takes into account the generation time, incubation period, and reporting delay directly. Firstly, the reproduction number is related to the observed growth rate through the generation interval distribution [69]. For each subsequent time step, the previous imputed infections are summed, weighted by an uncertain generation time probability mass function, and combined with an estimate of the $R_t$ to give the incidence [36, 45]. Biases in the $R_t$ estimate from misspecification of the generation interval is a significant source of over- or underestimation when the true values is significantly greater than or less than 1 [33]. $R_t$ estimates are affected by the mean generation time as well as the variance and shape of the generation interval distribution [33]. The intrinsic generation interval is used to link the $R_t$ and incidence of infections [69]. However, the intrinsic generation interval is rarely observed in the real world. Therefore, the generation interval is estimated from the serial interval [33].

Secondly, the infection trajectories are mapped to mean reported case counts using the incubation period and reporting delay distributions [36, 45]. If the distribution of the incubation period is known, the time lag between infection and symptom onset is taken into account [70]. The uncertainty of the reproduction number is exacerbated with significant variability of the time between infection and symptom onset [70].

Finally, time-series data of the number of new infections is used to estimate the $R_t$. These values are inferred based on assumptions regarding the delay time between infection and onset of symptoms. The accuracy is improved by inferring the unlagged time series of infections or within an $R_t$ estimation model [33]. The time series could simply be shifted by the mean delay to onset of symptoms, but would be significantly less accurate. If the delay to onset of symptoms is constant and the mean delay is known, then the shift of the observed time series is sufficient. However, the shift would not take into account the uncertainty or individual variation in delay times [33].

We sample from the delay distribution to impute individual times of infection from times of observation. However, the sampling strategy can smooth the underlying incidence curve and limit the ability of the estimates and predictions to rapidly reflect instantaneous changes in $R_t$ [33]. The present model can be modified to account for changes in the delay from symptom onset to notification about cases over the course of an outbreak. Additional data are needed for this purpose [36].

Delay distributions, interval specification, and truncation are required to infer the infection time series from recent observations and describe epidemic dynamics [33]. The workflow can explore how outbreak dynamics differ between particular sub-populations such as high-risk COVID-19 patients and how it can bias $R_t$ [36]. The current model can be used to characterize the relationship between a primary and secondary observation. For example, hospital admissions can be used to predict deaths or bed occupancy. Alternative model specifications can expand the scope of the study. A wide range of distributions and link functions is supported together with modeling options including non-linear and smoothing terms in addition to auto-correlation structures [46]. This results in a flexible modeling approach.

The Bayesian regression model includes region, day of the week, and date smoothed for seasonal patterns. The models can be extended to account for other cofactors or comorbidities adjusting for previously described risk factors for severe diseases. For example, age, sex, race, obesity, hypertension, arrhythmia, metabolic syndrome, cardiovascular disease, and chronic respiratory disease have been association with hospitalization [71]. Understanding risk factors for hospitalization helps facilitate targeted prevention messaging and forecasting and prioritization of clinical and public health resource needs [71]. Bayesian regression models can be extended to other problem settings and model systems in addition to different health outcomes and risk factors for new, emerging infectious diseases such as SARS, H1N1, and Ebola in West Africa [72–75].

Alternative implementations of the current model can estimate and forecast cases and hospitalizations for COVID-19. For example, the R package *EpiNow2* estimates a secondary observation from a primary observation correcting for delay distributions and truncation of data [45]. Similarly, the R package *bsts* samples from the posterior distribution of a Bayesian structural time series model while accounting for predictor variables [76–78]. The *bsts* model uses the local linear trend, seasonal pattern, and regression component. For the purposes of the workflow presented in this study, the R package *brms* implements a wide range of distributions and link functions in a multilevel context [46]. Autocorrelation of the response variable and user-defined covariance structures represent other options. Prior specifications of covariates of the brms models are flexible and model fits can easily be assessed [46]. *brms* allows the user to benefit from *Stan* for great modeling flexibility while using a simple, *lme4*-like formula syntax.

There are limitations on the quality and availability of the data and the assumptions of the model. COVID-19 data still underestimate the true burden of the epidemic in some counties, particularly in those with limited resources. Similarly, there is still limited information to determine the exact lag time in incubation period, reporting delay, and testing delay, particularly for counties where data are made available infrequently [79]. The use of reported cases as an input is an ongoing issue for assessing the quality of control measures and the tracing of epidemics where the frequency of asymptomatic infections, non-specific symptoms of mild disease, and limitations in testing capacity can lead to underreporting [80–83]. Consequently, parameter estimates and forecasts for models based on case data can be susceptible to uncertainty.

## Conclusions

We present an approach to estimate and forecast cases and hospitalizations for COVID-19 in addition to the corresponding uncertainty while using publicly available data. The workflow is able to infer short-term trends consistent with reported cases and hospitalizations at the HERC region level. Additionally, the workflow is able to accurately forecast and estimate the uncertainty of the measurements and provided Bayesian credible intervals. However, all three credible intervals of the three time horizons underestimate the true coverage probabilities for all three metrics and at least one of the horizons. We conclude that the uncertainty quantification should be reformulated as the frequentist coverage probability of the Bayesian credible interval.

Currently, the workflow generates daily updates about cases and hospitalizations to support decision making processes. The resulting tool assists in the containment of public health crises and aids in implementation of public health interventions. This workflow can be applied and automated to provide information regarding the growth and spread of COVID-19. The model anticipates surges of cases and hospitalizations a few critical days in advance. It has many advantages for those deciding where to direct prevention implementations and response improvements. We believe this study elucidates the geographical regions are most affected and the regions will encounter outbreaks.

## Data Availability

Data for COVID-19 confirmed cases and hospital admissions are publicly available and openly accessible from the Wisconsin Department of Health Services at https://data.dhsgis.wi.gov/datasets/covid-19-data-by-county-v2.

## Abbreviations

COVID-19: Coronavirus Disease 2019
SARS-CoV-2: Severe Acute Respiratory Syndrome-CoronaVirus-2
EM: Emergency Medicine
DHS: Department of Health Services
WEDSS: Wisconsin Electronic Disease Surveillance System
CDC: Centers for Disease Control and Prevention
HERC: Healthcare Emergency Readiness Coalition
CrI: Credible Interval

## References

[1] The New York Times. Coronavirus (Covid-19) Data in the United States. 2020. https://www.nytimes.com/interactive/2020/us/coronavirus-us-cases.html. Accessed 1 Nov 2021.

[2] The New York Times. We’re Sharing Coronavirus Case Data for Every U.S. County. 2020. https://github.com/nytimes/covid-19-data. Accessed 1 Nov 2021.

[3] Center for Systems Science and Engineering (CSSE) at Johns Hopkins University (JHU). COVID-19 Dashboard. 2020. https://coronavirus.jhu.edu/map.html. Accessed 1 Nov 2021.

[4] The New York Times. Coronavirus World Map: Tracking the Global Outbreak. 2020. https://www.nytimes.com/interactive/2020/world/coronavirus-maps.html. Accessed 1 Nov 2021.

[5] Johns Hopkins University (JHU). COVID-19 United States Cases by County. 2020. https://coronavirus.jhu.edu/us-map. Accessed 1 Nov 2021.

[6] World Health Organization. WHO Coronavirus (COVID-19) Dashboard. 2020. https://covid19.who.int/. Accessed 1 Nov 2021.

[7] The BBC Visual and Data Journalism Team. Covid map: Coronavirus cases, deaths, vaccinations by country. 2020. https://www.bbc.com/news/world-51235105. Accessed 1 Nov 2021.

[8] Beltrán ETM, Pérez MQ, Pastor-Galindo J, Nespoli P, Clemente FJG, Mármol FG (2021). COnVIDa: COVID-19 multidisciplinary data collection and dashboard. J Biomed Inform..

[9] Olshen AB, Garcia A, Kapphahn KI, Weng Y, Wesson PD, Rutherford GW, et al. COVIDNearTerm: A Simple Method to Forecast COVID-19 Hospitalizations. medRxiv. 2021. p. 2021.10.08.21264785. 10.1101/2021.10.08.21264785. 10.1017/cts.2022.389 PMC9161046 35720970

[10] Xie Y, Kulpanowski D, Ong J, Nikolova E, Tran NM. Predicting Covid-19 emergency medical service incidents from daily hospitalisation trends. Int J Clin Pract. 2021. p. e14920. 10.1111/ijcp.14920. 10.1111/ijcp.14920 34569674

[11] Putra M, Kesavan M, Brackney K, Hackney DN, Roosa KM (2020). Forecasting the impact of coronavirus disease during delivery hospitalization: an aid for resource utilization. Am J Obstet Gynecol MFM..

[12] Aviv-Sharon E, Aharoni A (2020). Generalized logistic growth modeling of the COVID-19 pandemic in Asia. Infect Dis Model..

[13] He S, Peng Y, Sun K (2020). SEIR modeling of the COVID-19 and its dynamics. Nonlinear Dyn..

[14] Rǎdulescu A, Williams C, Cavanagh K (2020). Management strategies in a SEIR-type model of COVID 19 community spread. Sci Rep..

[15] Morozova O, Li ZR, Crawford FW. One year of modeling and forecasting COVID-19 transmission to support policymakers in Connecticut. medRxiv. 2021. p. 2020.06.12.20126391. 10.1101/2020.06.12.20126391. 10.1038/s41598-021-99590-5 PMC8511264 34642405

[16] van Wees JD, Osinga S, van der Kuip M, Tanck M, Hanegraaf M, Pluymaekers M, et al. Forecasting hospitalization and ICU rates of the COVID-19 outbreak: an efficient SEIR model. 2020. 10.2471/BLT.20.256743.

[17] Arslan S, Ozdemir MY, Ucar A (2021). Nowcasting and Forecasting the Spread of COVID-19 and Healthcare Demand in Turkey, a Modeling Study. Front Public Health..

[18] Albani VVL, Velho RM, Zubelli JP (2021). Estimating, monitoring, and forecasting COVID-19 epidemics: a spatiotemporal approach applied to NYC data. Sci Rep..

[19] Keeling MJ, Hill EM, Gorsich EE, Penman B, Guyver-Fletcher G, Holmes A (2021). Predictions of COVID-19 dynamics in the UK: Short-term forecasting and analysis of potential exit strategies. PLOS Comput Biol..

[20] Carcione JM, Santos JE, Bagaini C, Ba J. A Simulation of a COVID-19 Epidemic Based on a Deterministic SEIR Model. Front Public Health. 2020;8:00230. 10.3389/fpubh.2020.00230. 10.3389/fpubh.2020.00230 PMC7270399 32574303

[21] Hethcote HW (2000). The Mathematics of Infectious Diseases. SIAM Rev..

[22] Zhang LJ, Li Y, Ren Q, Huo Z. Global Dynamics of an SEIRS Epidemic Model with Constant Immigration and Immunity. WSEAS Trans Math. 2013;12(5):11.

[23] Anastassopoulou C, Russo L, Tsakris A, Siettos C (2020). Data-based analysis, modelling and forecasting of the COVID-19 outbreak. PLoS ONE..

[24] Bastos SB, Cajueiro DO (2020). Modeling and forecasting the early evolution of the Covid-19 pandemic in Brazil. Sci Rep..

[25] Prem K, Liu Y, Russell TW, Kucharski AJ, Eggo RM, Davies N (2020). The effect of control strategies to reduce social mixing on outcomes of the COVID-19 epidemic in Wuhan, China: a modelling study. Lancet Public Health..

[26] Wang K, Lu Z, Wang X, Li H, Li H, Lin D (2020). Current trends and future prediction of novel coronavirus disease (COVID-19) epidemic in China: a dynamical modeling analysis. Mathematical Biosciences and Engineering..

[27] Moein S, Nickaeen N, Roointan A, Borhani N, Heidary Z, Javanmard SH (2021). Inefficiency of SIR models in forecasting COVID-19 epidemic: a case study of Isfahan. Sci Rep..

[28] Reiner RC, Barber RM, Collins JK, Zheng P, Adolph C, Albright J (2021). Modeling COVID-19 scenarios for the United States. Nat Med..

[29] Ioannidis JPA, Cripps S, Tanner MA. Forecasting for COVID-19 has failed. Int J Forecast. 2020. 10.1016/j.ijforecast.2020.08.004. 10.1016/j.ijforecast.2020.08.004 PMC7447267 32863495

[30] Liu L, Moon HR, Schorfheide F (2020). Forecasting With Dynamic Panel Data Models. Econometrica..

[31] Brown LD, Greenshtein E (2009). Nonparametric empirical Bayes and compound decision approaches to estimation of a high-dimensional vector of normal means. Ann Stat..

[32] Liu L, Moon HR, Schorfheide F (2021). Panel forecasts of country-level Covid-19 infections. J Econ..

[33] Gostic KM, McGough L, Baskerville EB, Abbott S, Joshi K, Tedijanto C (2020). Practical considerations for measuring the effective reproductive number, Rt. PLoS Comput Biol..

[34] Ganyani T, Kremer C, Chen D, Torneri A, Faes C, Wallinga J (2020). Estimating the generation interval for coronavirus disease (COVID-19) based on symptom onset data, March 2020. Eurosurveillance..

[35] Lauer SA, Grantz KH, Bi Q, Jones FK, Zheng Q, Meredith HR (2020). The Incubation Period of Coronavirus Disease 2019 (COVID-19) From Publicly Reported Confirmed Cases: Estimation and Application. Ann Intern Med..

[36] Abbott S, Hellewell J, Thompson R, Sherratt K, Gibbs H, Bosse N (2020). Estimating the time-varying reproduction number of SARS-CoV-2 using national and subnational case counts. Wellcome Open Res..

[37] Wisconsin Department of Health Services. COVID-19 Historical Data by County. 2020. https://data.dhsgis.wi.gov/datasets/covid-19-data-by-county. Accessed 1 Feb 2022.

[38] National Notifiable Diseases Surveillance System. Coronavirus Disease 2019 (COVID-19): 2020 Interim Case Definition, Approved August 5, 2020. 2020. https://wwwn.cdc.gov/nndss/conditions/coronavirus-disease-2019-covid-19/case-definition/2020/08/05/. Accessed 1 Nov 2020.

[39] Randhawa AK, Fisher LH, Greninger AL, Li SS, Andriesen J, Corey L (2020). Changes in SARS-CoV-2 Positivity Rate in Outpatients in Seattle and Washington State, March 1-April 16, 2020. JAMA..

[40] Gu Y. Estimating True Infections Revisited: A Simple Nowcasting Model to Estimate Prevalent Cases in the US. 2020. https://covid19-projections.com/estimating-true-infections-revisited/#adjusted-test-positivity. Accessed 1 Nov 2020.

[41] Ellis P. Test positivity rates and actual incidence and growth of diseases. 2020. http://freerangestats.info/blog/2020/05/09/covid-population-incidence. Accessed 1 Nov 2020.

[42] Ellis P. Incidence of COVID-19 in Texas after adjusting for test positivity. 2020. http://freerangestats.info/blog/2020/05/17/covid-texas-incidence. Accessed 1 Nov 2020.

[43] Ellis P. Estimating Covid-19 reproduction number with delays and right-truncation. 2020. http://freerangestats.info/blog/2020/07/18/victoria-r-convolution. Accessed 1 Nov 2020.

[44] Wisconsin Department of Health Services. Wisconsin Healthcare Emergency Preparedness Program. 2014. https://www.dhs.wisconsin.gov/preparedness/healthcare/index.htm. Accessed 1 Nov 2021.

[45] Abbott S, Hellewell J, Thompson R, Gostic K, Sherratt K, Meakin S, et al. EpiNow2: Estimate Real-Time Case Counts and Time-Varying Epidemiological Parameters. 2020. R package version 1.3.2. https://CRAN.R-project.org/package=EpiNow2. Accessed 14 Dec 2020.

[46] Bürkner PC (2017). brms: An R Package for Bayesian Multilevel Models Using Stan. J Stat Softw..

[47] Kleijn BJK. The frequentist theory of Bayesian statistics. 2nd ed. New York, NY: Springer-Verlag New York; 2020.

[48] R Core Team. R: A Language and Environment for Statistical Computing. Vienna; 2020. https://www.R-project.org/. Accessed 10 Oct 2020.

[49] Wickham H, Averick M, Bryan J, Chang W, McGowan LD, François R (2019). Welcome to the tidyverse. J Open Source Softw..

[50] Wood SN. Generalized Additive Models: An Introduction with R. 2nd ed. New York, NY: Chapman and Hall/CRC; 2017.

[51] Bürkner PC (2018). Advanced Bayesian Multilevel Modeling with the R Package brms. R J..

[52] Wickham H. ggplot2: Elegant Graphics for Data Analysis. Springer-Verlag New York; 2016. https://ggplot2.tidyverse.org. Accessed 25 Jun 2021.

[53] Sievert C. Interactive Web-Based Data Visualization with R, plotly, and shiny. Chapman and Hall/CRC; 2020. https://plotly-r.com. Accessed 10 Jan 2021.

[54] Hafen R. geofacet: ‘ggplot2’ Faceting Utilities for Geographical Data. 2020. R package version 0.2.0. https://CRAN.R-project.org/package=geofacet. Accessed 26 May 2020.

[55] Aravamuthan S, Mandujano Reyes J, Döpfer D. COVID-19 Cases, Effective R_t, and Hospitalizations: Geofacet by Wisconsin HERC Regions. 2021. https://data-viz.it.wisc.edu/cases-r-hosp-geofacet-wi-region/. Accessed 1 Feb 2021.

[56] Centers for Disease Control and Prevention. Cases in the US. 2020. https://www.cdc.gov/coronavirus/2019-ncov/cases-updates/cases-in-us.html. Accessed 1 Nov 2021.

[57] Afzal S, Ghani S, Jenkins-Smith HC, Ebert DS, Hadwiger M, Hoteit I. A Visual Analytics Based Decision Making Environment for COVID-19 Modeling and Visualization. 2020. arXiv:2010.11897.

[58] Zhigljavsky A, Fesenko I, Wynn H, Whitaker R, Kremnizer K, Noonan J, et al. A prototype for decision support tool to help decision-makers with the strategy of handling the COVID-19 UK epidemic. medRxiv. 2020. 10.1101/2020.04.24.20077818.

[59] Yang C, Zhang Z, Fan Z, Jiang R, Chen Q, Song X, et al. EpiMob: Interactive Visual Analytics of Citywide Human Mobility Restrictions for Epidemic Control. 2021. arXiv:2007.03180. 10.1109/TVCG.2022.3165385 35385385

[60] Yañez A, Duggan J, Hayes C, Jilani M, Connolly M. PandemCap: Decision support tool for epidemic management. In: 2017 IEEE Workshop on Visual Analytics in Healthcare (VAHC). 2017. p. 24–30. 10.1109/VAHC.2017.8387497.

[61] Lutz CS, Huynh MP, Schroeder M, Anyatonwu S, Dahlgren FS, Danyluk G (2019). Applying infectious disease forecasting to public health: a path forward using influenza forecasting examples. BMC Public Health..

[62] Doms C, Kramer SC, Shaman J (2018). Assessing the Use of Influenza Forecasts and Epidemiological Modeling in Public Health Decision Making in the United States. Sci Rep..

[63] Moss R, Fielding JE, Franklin LJ, Stephens N, McVernon J, Dawson P (2018). Epidemic forecasts as a tool for public health: interpretation and (re)calibration. Aust N Z J Public Health..

[64] Funk S, Camacho A, Kucharski AJ, Lowe R, Eggo RM, Edmunds WJ (2019). Assessing the performance of real-time epidemic forecasts: A case study of Ebola in the Western Area region of Sierra Leone, 2014–15. PLOS Comput Biol..

[65] Dunson DB (2001). Commentary: Practical Advantages of Bayesian Analysis of Epidemiologic Data. Am J Epidemiol..

[66] Tolles J, Luong T (2020). Modeling Epidemics With Compartmental Models. JAMA..

[67] Sherratt K, Abbott S, Meakin SR, Hellewell J, Munday JD, Bosse N (1829). Exploring surveillance data biases when estimating the reproduction number: with insights into subpopulation transmission of COVID-19 in England. Phil Trans R Soc B Biol Sci..

[68] Bracher J, Wolffram D, Deuschel J, Görgen K, Ketterer JL, Ullrich A (2021). A pre-registered short-term forecasting study of COVID-19 in Germany and Poland during the second wave. Nat Commun..

[69] Wallinga J, Lipsitch M (2007). How generation intervals shape the relationship between growth rates and reproductive numbers. Proc R Soc B Biol Sci..

[70] Fraser C (2007). Estimating Individual and Household Reproduction Numbers in an Emerging Epidemic. PLoS ONE..

[71] Vahey GM, McDonald E, Marshall K, Martin SW, Chun H, Herlihy R (2021). Risk factors for hospitalization among persons with COVID-19-Colorado. PLoS ONE..

[72] Cauchemez S, Boelle PY, Donnelly CA, Ferguson NM, Thomas G, Leung GM (2006). Real-time estimates in early detection of SARS. Emerg Infect Dis..

[73] Bauch CT, Lloyd-Smith JO, Coffee MP, Galvani AP. Dynamically Modeling SARS and Other Newly Emerging Respiratory Illnesses: Past, Present, and Future. Epidemiology. 2005;16(6):791–801. https://www.jstor.org/stable/20486145. 10.1097/01.ede.0000181633.80269.4c 16222170

[74] Biggerstaff M, Cauchemez S, Reed C, Gambhir M, Finelli L (2014). Estimates of the reproduction number for seasonal, pandemic, and zoonotic influenza: a systematic review of the literature. BMC Infect Dis..

[75] Althaus CL, Estimating the Reproduction Number of Ebola Virus (EBOV) During the 2014 Outbreak in West Africa. PLoS Curr Outbreaks. 2014. 10.1371/currents.outbreaks.91afb5e0f279e7f29e7056095255b288. 10.1371/currents.outbreaks.91afb5e0f279e7f29e7056095255b288 PMC4169395 25642364

[76] Scott SL. bsts: Bayesian Structural Time Series. 2020. R package version 0.9.5. https://CRAN.R-project.org/package=bsts.

[77] Scott SL, Varian HR (2014). Predicting the present with Bayesian structural time series. Int J Math Model Numer Optimisation..

[78] Scott SL. Fitting Bayesian structural time series with the bsts R package. 2014. https://www.unofficialgoogledatascience.com/2017/07/fitting-bayesian-structural-time-series.html. Accessed 1 Nov 2021.

[79] Harris JE (2022). Timely epidemic monitoring in the presence of reporting delays: anticipating the COVID-19 surge in New York City, September 2020. BMC Public Health..

[80] Mizumoto K, Kagaya K, Zarebski A, Chowell G (2020). Estimating the asymptomatic proportion of coronavirus disease 2019 (COVID-19) cases on board the Diamond Princess cruise ship, Yokohama, Japan, 2020. Eurosurveillance..

[81] Wynants L, Calster BV, Collins GS, Riley RD, Heinze G, Schuit E (2020). Prediction models for diagnosis and prognosis of covid-19: systematic review and critical appraisal. BMJ..

[82] Russell TW, Golding N, Hellewell J, Abbott S, Wright L, Pearson CAB, et al. Reconstructing the early global dynamics of under-ascertained COVID-19 cases and infections. medRxiv. 2020. 10.1101/2020.07.07.20148460. 10.1186/s12916-020-01790-9 PMC7577796 33087179

[83] Whittaker C, Walker PGT, Alhaffar M, Hamlet A, Djaafara BA, Ghani A (2021). Under-reporting of deaths limits our understanding of true burden of covid-19. BMJ..

